# Sarmentine, a natural herbicide from *Piper* species with multiple herbicide mechanisms of action

**DOI:** 10.3389/fpls.2015.00222

**Published:** 2015-04-08

**Authors:** Franck E. Dayan, Daniel K. Owens, Susan B. Watson, Ratnakar N. Asolkar, Louis G. Boddy

**Affiliations:** ^1^Natural Products Utilization Research Unit, US Department of Agriculture-Agricultural Research Service, Thad Cochran Center, University of MississippiMS, USA; ^2^Marrone Bio InnovationsDavis, CA, USA

**Keywords:** mode of action, photosystem II, electrolyte leakage, membrane integrity, enoyl ACP reductase, herbicide resistance, herbicide discovery

## Abstract

Sarmentine, 1-(1-pyrrolidinyl)-(2*E*,4*E*)-2,4-decadien-1-one, is a natural amide isolated from the fruits of *Piper* species. The compound has a number of interesting biological properties, including its broad-spectrum activity on weeds as a contact herbicide. Initial studies highlighted a similarity in response between plants treated with sarmentine and herbicidal soaps such as pelargonic acid (nonanoic acid). However, little was known about the mechanism of action leading to the rapid desiccation of foliage treated by sarmentine. In cucumber cotyledon disc-assays, sarmentine induced rapid light-independent loss of membrane integrity at 100 μM or higher concentration, whereas 3 mM pelargonic acid was required for a similar effect. Sarmentine was between 10 and 30 times more active than pelargonic acid on wild mustard, velvetleaf, redroot pigweed and crabgrass. Additionally, the potency of 30 μM sarmentine was greatly stimulated by light, suggesting that this natural product may also interfere with photosynthetic processes. This was confirmed by observing a complete inhibition of photosynthetic electron transport at that concentration. Sarmentine also acted as an inhibitor of photosystem II (PSII) on isolated thylakoid membranes by competing for the binding site of plastoquinone. This can be attributed in part to structural similarities between herbicides like sarmentine and diuron. While this mechanism of action accounts for the light stimulation of the activity of sarmentine, it does not account for its ability to destabilize membranes in darkness. In this respect, sarmentine has some structural similarity to crotonoyl-CoA, the substrate of enoyl-ACP reductase, a key enzyme in the early steps of fatty acid synthesis. Inhibitors of this enzyme, such as triclosan, cause rapid loss of membrane integrity in the dark. Sarmentine inhibited the activity of enoyl-ACP reductase, with an *I*_50_app of 18.3 μM. Therefore, the herbicidal activity of sarmentine appears to be a complex process associated with multiple mechanisms of action.

## Introduction

Herbicides benefit food production by reducing weed pressure and improving the quality of crop products (Gianessi and Reigner, 2007). However, concerns over their potential adverse effect on the environment and human health are leading consumers to desire agricultural crops produced with greener technologies (Solomon and Schettler, 2000; Stillerman et al., 2008). Additionally, reliance on herbicides has led to the emergence of resistance among many weed species (Délye et al., 2013; Service, 2013; Heap, 2015). Therefore, there is a need to develop alternative ecofriendly, economical and efficacious means for weed management. To that end, there is a renewed interest in discovering and developing new biopesticides (Cantrell et al., 2012; Dayan et al., 2012).

Bioherbicides, such as pelargonic acid, are non-selective and less potent than their synthetic counterparts, requiring multiple applications at relatively high concentrations (Young, 2004; Barker and Prostak, 2009). However, some natural products have served as templates for the development of several successful herbicides (glufosinate and the triketone herbicides) (Cantrell et al., 2012) that introduced new mechanisms of action, which is greatly needed to combat the evolution of resistance to herbicides in production agriculture (Duke et al., 2013). There is, therefore, a great interest in exploring natural products to develop new bioherbicides (Hüter, 2011; Dayan et al., 2012; Gerwick and Sparks, 2014). Natural products are particularly attractive as templates because they occupy a wider chemical space with greater structural diversity than traditional synthetic compounds (Koch et al., 2005; Lipkus et al., 2008; Li and Vederas, 2009; Valli et al., 2013).

*Piper* species produce a large number of bioactive molecules with great economic value (Parmar et al., 1997). Yet, this may only represent a small portion of the true breadth of the chemical richness of *Piper* metabolites since only a limited number of *Piper* species have been characterized (Dyer et al., 2004). *Piper* amides (e.g., piperidine amides) are a particularly prevalent group of neutral to weakly acidic bioactive products (Likhitwitayawuid et al., 1987; Banerji and Das, 1989), with medicinal (Wang et al., 2014), insecticidal (Yang et al., 2002; Scott et al., 2008), antifungal (Alécio et al., 1998; Da Silva et al., 2014), and antiherbivory activity (Dyer et al., 2001).

Sarmentine, 1-(1-pyrrolidinyl)-(2*E*,4*E*)-2,4-decadien-1-one, is a *Piper* amide with several biological properties, including antiplasmodial, antimycobacterial, and antituberculosis activity (Rukachaisirikul et al., 2004; Tuntiwachwuttikul et al., 2006), as well as antiplatelet aggregation (Li et al., 2007). Interest in sarmentine as a biopesticide renewed when its herbicidal activity was discovered through a bioactivity-guided isolation of the active components of the fruits of *Piper sarmentosum* and *Piper nigrum* (Huang et al., 2010). Sarmentine acts as a contact herbicide with broad-spectrum activity in a similar way as herbicidal soaps such as nonanoic acid (pelargonic acid) or decanoic acid.

Most natural contact herbicides (pelargonic acid, essential oils) are used as desiccants and act by stripping the cuticular wax layer covering the surface of leaves (Fukuda et al., 2004; Coleman and Penner, 2006, 2008). The rapid desiccation of foliage treated by sarmentine suggests that this *Piper* amide has a similar mechanism of action. However, evidence that sarmentine acted in this way was lacking and this study investigates the mechanism of action of this biopesticide using methods developed in our laboratory (Dayan et al., 2000a, 2015).

## Materials and method

### Isolation and weed spectrum of sarmentine

Sarmentine was purified from long pepper fruit according to a protocol developed previously (Huang et al., 2010). All seedlings of weeds were planted in 5.7 × 5.7 × 6.2 cm or 8 × 8 × 7.2 cm plastic pots. All pots were stored in a growth room with 28°C temperature and 60% humidity. Seedlings including the broadleaf weeds wild mustard [*Brassica kaber* (DC.) L.C. Wheeler], pigweed (*Amaranthus retroflexus* L.), lambsquarters (*Chenopodium album* L.), velvetleaf (*Abutilon theophrasti* Medik.), common purslane (*Portulaca oleracea* L.), dandelion (*Taraxacum officinale* G.H. Weber ex Wiggers), bindweed (*Convolvulus arvensis* L.), spurge (*Euphorbia* sp.), common chickweed [*Stellaria media* (L.) Vill.], coffeeweed [*Sesbania exaltata* (Raf.) Rydb. ex A.W. Hill], white clover (*Trifolium repens* L.) and buckhorn plantain (*Plantago lanceolata* L.), and monocotyledonous weeds smallflower umbrella sedge (*Cyperus difformis* L.), goosegrass [*Eleusine indica* (L.) Gaertn.], large crabgrass [*Digitaria sanguinalis* (L.) Scop], annual bluegrass (*Poa annua* L.), late watergrass [*Echinochloa phyllopogon* (Stapf) Koss], quackgrass [*Elytrigia repens* (L.) Gould], smooth crabgrass [*Digitaria ischaemum* (Schreb.) Schreb. ex Muhl.] and yellow nutsedge (*Cyperus esculentus* L.), were planted in potting soil mixture. When treated, all seedlings were at the 2–3 true leaf stage. Visual injury was evaluated using the following rating scale: + = 0–50%, ++ = 51–80%, + + + = 81–90%, and + + ++ = 91–100% injury.

Plants in the growth room were treated with formulated sarmentine. The proprietary formulation consists of 50% sarmentine, with the remainder comprised of inert ingredients; it does not include any other herbicidal compounds. Pots were sprayed with the equivalent of 10 mg sarmentine ml^−1^ in water; no additional surfactant was added. Solutions were applied with a Generation III track sprayer (DeVries Manufacturing, Hollandale, MN, USA) equipped with a model TeeJet EZ 8002 nozzle (Spraying Systems Co., Wheaton, IL, USA) with conical pattern and 80° spray angle. The height from nozzle to soil level was 71 cm for the experiments. The spray head was set to move over the plants at 1.7 km h^−1^, and the sprayer was calibrated to deliver the equivalent of 374 l ha^−1^.

### Leaf surface microscopy

Leaves were collected from 2 to 3 week-old plants for scanning electron microscopy observation. To preserve the appearance of the cuticles, samples were processed without fixation and dehydration by flash-freezing in supercooled nitrogen slush at −210°C for 5 s according to Hayat (2000). The frozen samples were placed in 1 kg brass receptacles supercooled in liquid nitrogen (−196°C) to keep the leaves frozen during the initial stages of lyophilization at −50°C. The lyophilized leaf samples were then placed on aluminum stubs with their adaxial surfaces exposed and coated with a layer of gold-palladium (15 mA under 75 mTorr pressure) using a Hummer 6.2 sputter coater (Anatech USA, Union City, CA 94587). The adaxial surfaces of several samples of each species were observed with a scanning electron microscope (JEOL JSM-5600, Peabody, MA 01960). The images were digitally colorized using Adobe Photoshop CS4 (Adobe, San Jose, CA 95110).

### Electrolyte leakage

The effect of sarmentine and pelargonic acid on plasma membrane integrity was tested by measuring electrolyte leakage as described before (Dayan and Watson, 2011). Briefly, cucumber seedlings [*Cucumis sativus* (L.) var. straight eight] were maintained in a growth chamber with a 16/8 light/dark cycle for 10 days. Twenty-five 4-mm cotyledon discs were incubated in the presence of different concentrations of the sarmentine or pelargonic acid. Sarmentine and pelargonic acid stocks (100X) were made in acetone. Control tissues were exposed to the same amount of acetone (1% [v/v]) as treated tissues but without test compounds. Plates were incubated in darkness for 24 h prior to exposure to high light intensity (1000 μmol m^−2^ s^−1^) photosynthetically active radiation (PAR).

Measurements were made using an electrical conductivity meter (Model 1056, Amber Science, Eugene, OR 97402) equipped with a model 858 Conductivity Macro Flow cell. Measurements were taken at the start of the experiment, at the end of the dark incubation period, and after exposure to high light intensity. The experiment was repeated over time and consisted of three replicates.

Subsequent experiments with velvetleaf (*Abutilon theophrasti* Medik.), redroot pigweed (*Amaranthus retroflexus* L.), mustard [*Brassica juncea* (L.) Czern] and large crabgrass [*Digitaria sanguinalis* (L.) Scop.] consisted of a measurement of the conductivity in the bathing medium at the beginning of the experiment and after 24 h incubation in darkness.

### Induced chlorophyll fluorescence

The effect of sarmentine on photosynthesis was initially tested on cucumber cotyledons from the leakage experiments using a pulse-modulated fluorometer (Opti-Science, Model OS5-FL, Tyngsboro, MA 01879). The instrument was set on Kinetic Mode and adjusted so that the initial Ft (instantaneous fluorescence signal) value in the control samples was approximately 210. The other parameters were as described in a previous publication (Dayan and Zaccaro, 2012).

### Inhibition of oxygen evolution in isolated thylakoid membranes

Thylakoid membranes were isolated from spinach, or wild-type and triazine-resistant redroot pigweed (*Amaranthus retroflexus* L.) as described elsewhere (Rimando et al., 1998; Dayan et al., 2009a), except that the thylakoid membranes were further purified on a 30–52% sucrose step gradient centrifugation in a SW40 Ti swinging bucket rotor and an XL-90 Beckman (Beckman Coulter, Inc., Brea, CA 92821-6232 USA) centrifuge at 28,000 g for 1 h at 4°C (Dayan et al., 2009b). Thylakoid membranes were diluted to 4 mg of chlorophyll ml^−1^ for the oxygen evolution experiments and to 1 mg of chlorophyll ml^−1^ for the binding kinetic experiments.

### Oxygen evolution assay

O_2_ evolution assays were conducted under saturating light conditions (10 mmol m^−2^ s^−1^ PAR) with the use of a fiber-optic light source delivering 1300 lumen (Schott-Foster, LLC, Southbridge, MA 01550 USA) and measured using a Hansatech OXYGRAPH PLUS Oxygen Electrode System (PP System, Amesbury, MA 01913 USA) as described elsewhere (Dayan et al., 2009b). Sarmentine and atrazine were diluted in acetone, and control treatments received the same concentration of solvent (less than 1% v/v). Membrane preparations were incubated with test compounds (0–3 mM) on ice for 20 min prior to the assay. The assay was initiated by addition of thylakoid membranes to the reaction assay buffer, and the rate of oxygen evolution was measured for 120 s over the linear portion of the curve. Data are expressed as μmol O_2_ l^−1^ min^−1^.

### Binding kinetics of sarmentine on Q_B_ binding site of photosystem II

[^14^C]-atrazine was bound to spinach thylakoid membranes in the presence or absence of sarmentine according to Tischer and Strotmann (1977) as modified by Dayan et al. (2000b). Thylakoid membranes (100 μg of chlorophyll ml^−1^) were suspended in a 1-ml reaction solution consisting of 330 mM sorbitol, 100 mM HEPES (pH 7.7), 1 mM EDTA, and 1 mM MgCl_2_. A half-log dilution series (33–0.03 μM) of [^14^C]-atrazine (uniformly labeled with specific activity of 160 mCi mmol^−1^, American Radiolabeled Chemicals Inc, St. Louis, MO) and 10 μM unlabeled sarmentine was added. The suspensions were thoroughly mixed and incubated for 15 min on ice. The samples were centrifuged (6 min, 12,000g, 4°C). The supernatant was transferred to vials and mixed with 18 ml of premixed scintillation cocktail (Ultima Gold, Packard Instrument) for radioactivity measurements. The inner walls of tubes were dried with cotton swabs without disturbing the pellets to remove excess [^14^C]-atrazine. A 100-μl aliquot of tissue solubilizer (Soluene-350, Packard Instrument Co. Meriden, CT 06450) was added to the pellets and heated in a water bath at 50°C for 15 min. The slurry was neutralized with 50 μl of 1 M Tris-HCl buffer (pH 7.0) and transferred to scintillation vials. Ethanol (20 μl) used to wash the inner walls of each tube was combined with the slurry before radioactivity measurements. The amount of bound [^14^C]-atrazine was calculated from the radioactivity in the pellets. Binding for atrazine and sarmentine was determined from double-reciprocal plots of bound atrazine vs. free atrazine (Tischer and Strotmann, 1977). All regressions and intercepts were calculated in SigmaPlot (version 12, Systat Software Inc., San Jose, CA, USA).

### Inhibition of enoyl-ACP reductase (ENR)

*Arabidopsis thaliana* ENR was cloned in *Escherichia coli* and expressed as described elsewhere (Dayan et al., 2008). Briefly, cells were collected by centrifugation, resuspended in cold lysing buffer [50 mM, Tris-HCl (pH 7.5), 1 M NaCl, 5 mM, imidazole, 10% [v/v] glycerol, 1 μg ml^−1^ leupeptin] and lysed with a French Press (Glen Mills Inc., Clifton, NJ). Debris was removed by centrifugation and the ENR in the supernatant was purified on a nickel activated HisTrap HP column (GE Healthcare Bio-Sciences). The ENR containing fraction was desalted on a PD-10 column equilibrated with cold desalting buffer (10 mM sodium phosphate, pH 7.2, 10 mM dithiothreitol, 10% [v/v] glycerol). Protein concentration was determined using Bio-Rad protein reagent.

ENR activity was measured in 10 mM sodium phosphate, pH 7.2 assay buffer as described before (Dayan et al., 2008). The *I*_50_ values (concentration of inhibitor required for 50% inhibition of activity) of sarmentine, pelargonic acid, and triclosan on *A. thaliana* ENR were determined by testing the inhibitors at concentrations ranging from 0.1 to 100 μM. All inhibitors were dissolved in acetone and control samples received equivalent amounts of acetone (1% [v/v] final concentration). The reactions were started by addition of NADH. The oxidation of NADH was monitored for 60 s by measuring change in A340 in a Shimadzu model UV3101PC spectrophotometer with the cell thermostabilized at 25°C. Spectrophotometric measurements were converted to concentrations of NADH oxidized using ε = 6.3 mM^−1^ cm^−1^ (Ward et al., 1999).

### l-amino acid oxidase assay

All reagents, including the amino acid oxidase and peroxidase enzymes, were obtained from Sigma. The activity of l-amino acid oxidase was measured at 37°C using a Shimadzu spectrophotometer by measuring the increase in absorption at 436 nm via a peroxidase-coupled secondary reaction. The reaction buffer consisted of 0.2 M triethanolamine, pH 7.8 containing 0.1% l-leucine and 0.0065% *o*-dianisidine (Fast Blue B). Baseline conditions were established by adding 10 μl of a 10 mg ml^−1^ solution of peroxidase to 2.9 ml of reaction buffer in a cuvette. The cuvette was placed in the spectrophotometer and the temperature allowed to equilibrate for 5 min. The reaction was initiated by adding 100 μl of amino acid oxidase (0.027 units) to the cuvette and mixing. The amino acid oxidase was preincubated with 3 mM sarmentine or acetone for 15 min before measuring activity. The final concentration in the assay was 100 μM sarmentine.

### Computer modeling

Sarmentine and diuron were built using the fragment library of Spartan version 6.1 (Wavefunction, Inc., Irvine, CA) and minimized using the equilibrium geometry at ground state, by applying the semiempirical AM1 (Austin Model) parameterization starting with the MMFF (molecular mechanics force fields) geometry. Sarmentine and diuron were aligned along their amide moiety using the align function of Spartan. The π-charge distribution over the amide bond was visualized by applying the ionization calculation to the potential surface map.

### Statistical analysis

Dose-response curves were analyzed by a four-parameters log-logistic model (Seefeldt et al., 1995) using R software (version 2.15.2, R Foundation for Statistical Computing, Vienna, Austria) with the drc module (Ritz and Streibig, 2005). Means and standard deviations were obtained using the raw data and the half-maximal inhibitory response (*I*_50_) was defined as the concentration at which this accumulation was inhibited by 50% compared with controls. *I*_50_ values were obtained from the parameters in the regression curves. Graphs were generated with Sigma Plot. Means were separated with the Duncan multiple range test at *P* = 0.05 using the Agricolae module (De Mendiburu, 2014).

## Results

### Weed spectrum of sarmentine

Sarmentine was sprayed on selected broadleaf and grass weeds to determine its spectrum of activity. Wild mustard and pigweed were the most sensitive to sarmentine, with greater than 91% injury 7 days after treatment (Table 1). A number of broadleaf and grass weeds sustained between 81 and 90% injury (Tables 1, 2).

**Table 1:** **Herbicidal activity of sarmentine on selected broadleaf weeds 7 days after treatment**.

**Common name**	**Scientific name**	**Bayer code**	**Treatment (20 mg ml^−1^)^a^**
Wild mustard	*Brassica kaber*	SINAR	++++
Pigweed	*Amaranthus retroflexus*	AMARE	++++
Lambsquarters	*Chenopodium album*	CHEAL	+++
Velvetleaf	*Abutilon theophrasti*	ABUTH	+++
Common purslane	*Portulaca oleracea*	POROL	+++
Dandelion	*Taraxacum officinale*	TAROF	+++
Bindweed	*Convolvulus arvensis*	CONAR	+++
Spurge	*Euphorbia spathulata*	EPHSQ	++
Common chickweed	*Stellaria media*	STEME	++
Coffeeweed	*Sesbania exaltata*	SEBEX	++
White clover	*Trifolium repens*	TRFRE	+
Buckhorn plantain	*Plantago lanceolata*	PLALA	+

a *Ratings: + = 0–50%, ++ = 51–80%, +++ = 81–90%, and ++++ = 91–100% weed injury*.

**Table 2:** **Herbicidal activity of sarmentine on selected monocotyledonous weeds 7 days after treatment**.

**Common name**	**Scientific name**	**Bayer code**	**Treatment (20 mg ml^−1^)^a^**
Smallflower sedge	*Cyperus difformis*	CYPDI	+++
Goosegrass	*Eleusine indica*	ELEIN	++
Large crabgrass	*Digitaria sanguinalis*	DIGSA	++
Annual bluegrass	*Poa annua*	POAAN	++
Late watergrass	*Echinochloa phyllopogon*	ECHPH	+
Quackgrass	*Elytrigia repens*	AGRRE	+
Smooth crabgrass	*Digitaria ischaemum*	DIGIS	+
Yellow nutsedge	*Cyperus esculentus*	CYPES	+

a *Ratings: + = 0–50%, ++ = 51–80%, +++ = 81–90%, and ++++ = 91–100% weed injury*.

### Alteration of the leaf surface ultrastructure by sarmentine

**Figure 1:**
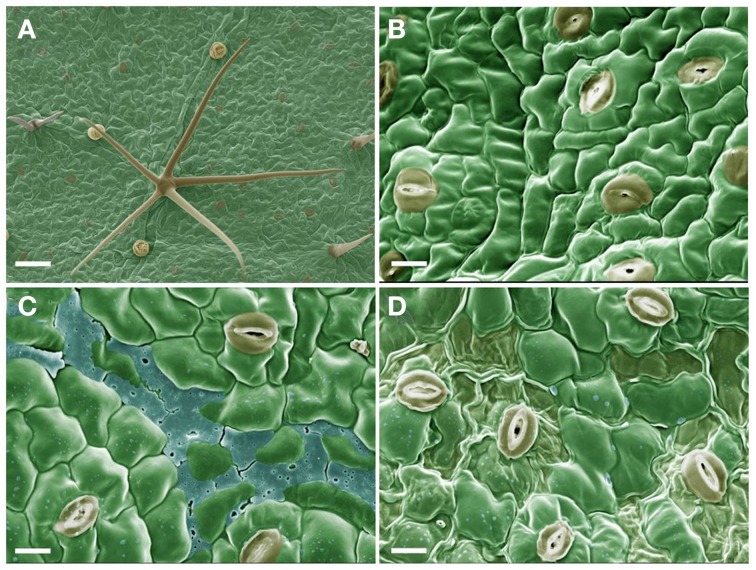
**Scanning electron micrographs of the adaxial leaf surface of velvetleaf. (A)** 30X magnification and bar = 100 μm. **(B–D)** are at 100X magnification and bar = 10 μm after 0, 1, and 4 h exposure to 1 mM sarmentine, respectively.

The adaxial leaf surface of velvetleaf consists of relatively smooth cuticle over the epidermal cells interspersed with stomata, glandular trichomes and star-shaped hairs over the surface (Figures 1A,B). The deposition of sarmentine over the leaf surface is visible within 1 h of application as a small residue scattered on the surfaces of epidermal cells as well as a layer accumulating in the grooves between the cells (Figure 1C). The desiccating effect of sarmentine is visible within 8 h of application as a localized loss of turgor of epidermal cells in areas where sarmentine has accumulated (Figure 1D).

### Destabilization of plasma membrane integrity

**Figure 2:**
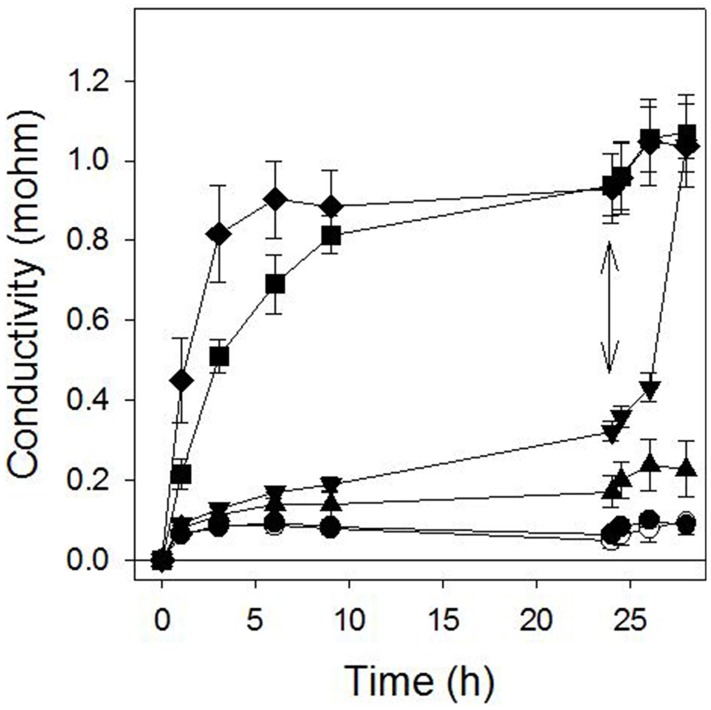
**Effect of sarmentine on cucumber membrane integrity as measured in the conductivity of the bathing medium**. Samples were incubated in darkness for 24 h and then exposed to high light intensity. ○ = control; ● = 3 μM; ▲ = 10 μM; ▼ = 30 μM; ■ = 100 μM; ♦ = 300 μM sarmentine. The double arrow marks when the samples were transferred to the light.

Sarmentine caused a light-independent loss of plasma membrane integrity in the cucumber cotyledon discs assays when tested at 100 μM or more. At these concentrations, a rapid increase in electrolyte conductivity was measured in the bathing medium within 1 h or exposure and reached a maximum between 6 and 8 h exposure (Figure 2). Interestingly, the activity of 30 μM sarmentine was low during the dark incubation period, but was greatly stimulated upon light exposure (double arrow on Figure 2).

**Figure 3:**
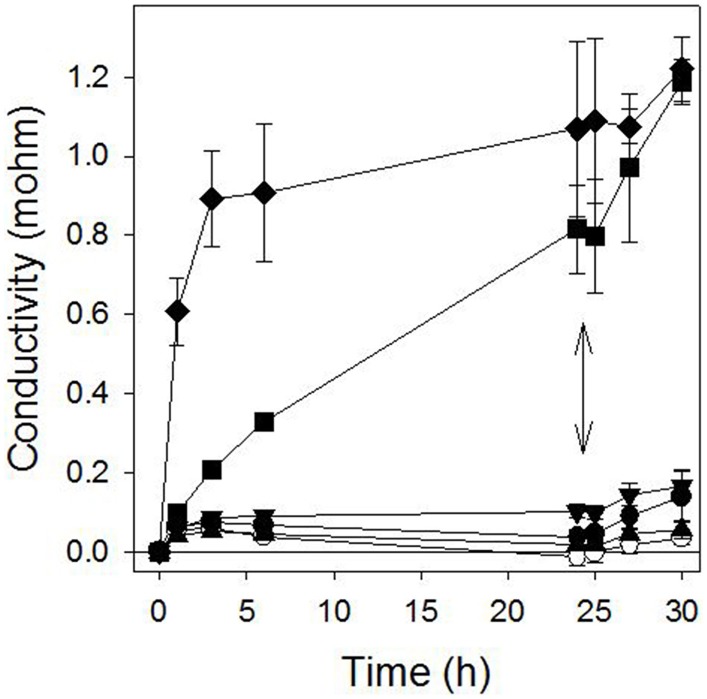
**Effect of pelargonic acid on cucumber membrane integrity as measured in the conductivity of the bathing medium**. Samples were incubated in darkness for 24 h and then exposed to high light intensity. ○ = control; ● = 30 μM; ▲ = 100 μM; ▼ = 300 μM;■ = 1000 μM; ♦ = 3000 μM pelargonic acid. The double arrow marks when the samples were transferred to the light.

**Figure 4:**
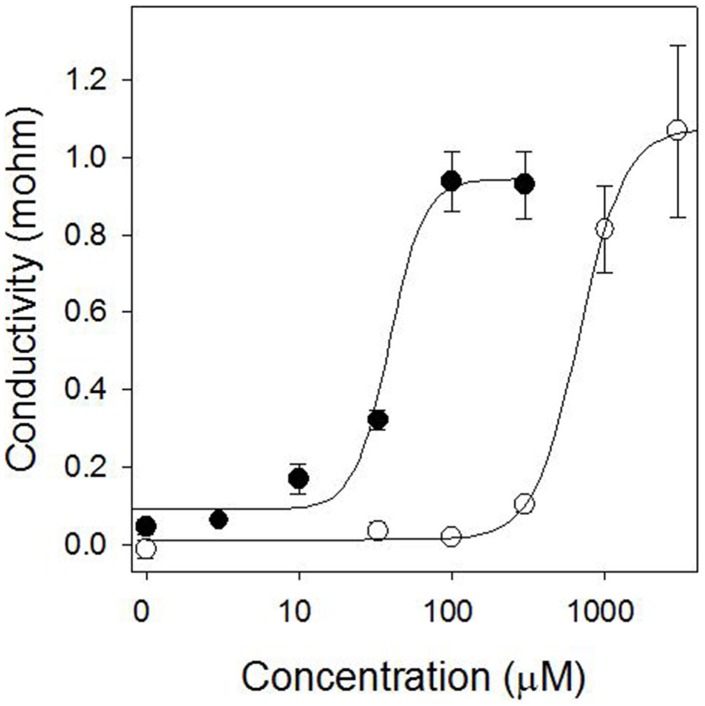
**Dose-response curve of sarmentine (●) and pelargonic acid (○) after 24 h incubation in the dark on membrane integrity in cucumber cotyledons**.

Pelargonic acid required higher concentrations to cause a similar light-independent loss of membrane integrity, and the activity was not stimulated by the addition of light (Figure 3). Plotting the relationship between inhibitor concentration and conductivity of the bathing solution after 24 h of incubation in darkness generated reliable dose-response curves that enabled the quantitative comparison of the potency of sarmentine and pelargonic acid (Figure 4).

Analysis of the curves were performed with a four-parameters log-logistic model which enabled the calculation of the *I*_50_ concentration for these compounds on various weed species (Table 3). Sarmentine was 12.4–29.1 times more active than pelargonic acid and had a more consistent potency across the species tested.

**Table 3:** **Effect of sarmentine and pelargonic acid on membrane integrity of cucumber, mustard, redroot pigweed, velvetleaf, crabgrass after 24 h incubation in darkness**.

Species	Sarmentine		Pelargonic acid		Potency^b^
	***I_50_* (μM)^a^**				
Cucumber	38.3 ± 4.5	A	645 ± 71	A	16.8
Redroot pigweed	38.3 ± 4.6	A	938 ± 72	A	24.5
Mustard	74.6 ± 6.7	B	926 ± 90	A	12.4
Velvetleaf	80.3 ± 3.9	B	2334 ± 423	B	29.1
Crabgrass	91.8 ± 9.4	C	2509 ± 191	B	27.3

a *Means values followed by the same letter do not differ significantly at the 5% level by Duncan's multiple range test*.

b *Potency of sarmentine relative to pelargonic acid: I_50_ of pelargonic acid/I_50_ of sarmentine*.

### Inhibition of enoyl-ACP reductase (ENR)

**Figure 5:**
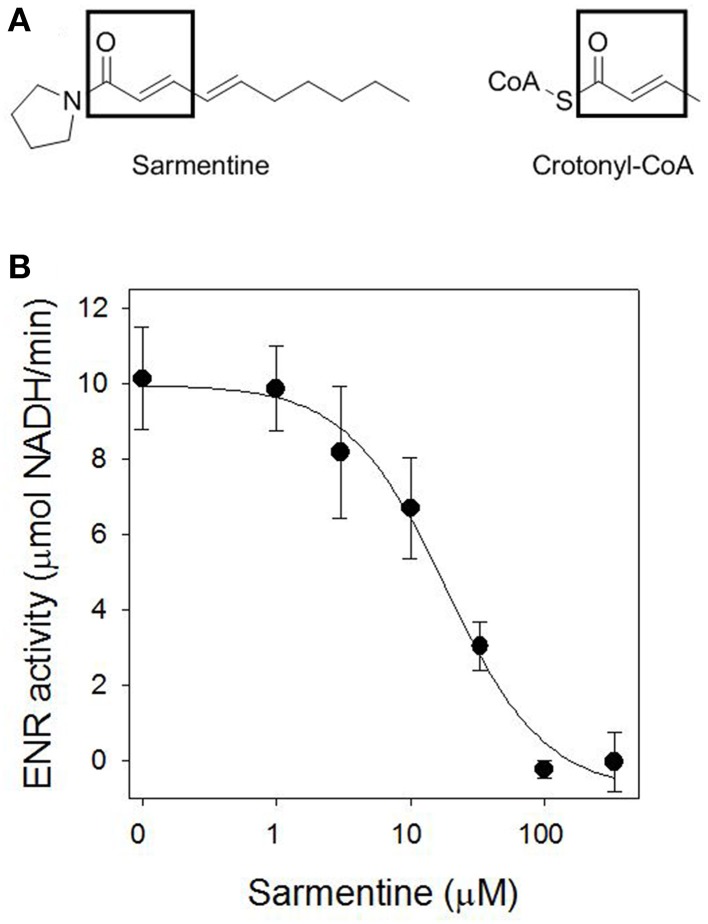
**(A)** Structure of sarmentine and crotonyl-CoA, and **(B)** effect of sarmentine on purified *Arabidopsis thaliana* enoyl-ACP reductase.

Sarmentine has some structural similarity with crotonyl-CoA, the substrate of ENR (Figure 5A). ENR was expressed heterologously in *E. coli* and purified with a specific activity of 10.13 ± 1.36 mmol NADH min^−1^ μg^−1^ protein. Sarmentine inhibited the activity of ENR in a dose-dependent manner with an *I*_50_app of 18.3 μM (Figure 5B).

### Interaction between sarmentine and photosystem II

**Figure 6:**
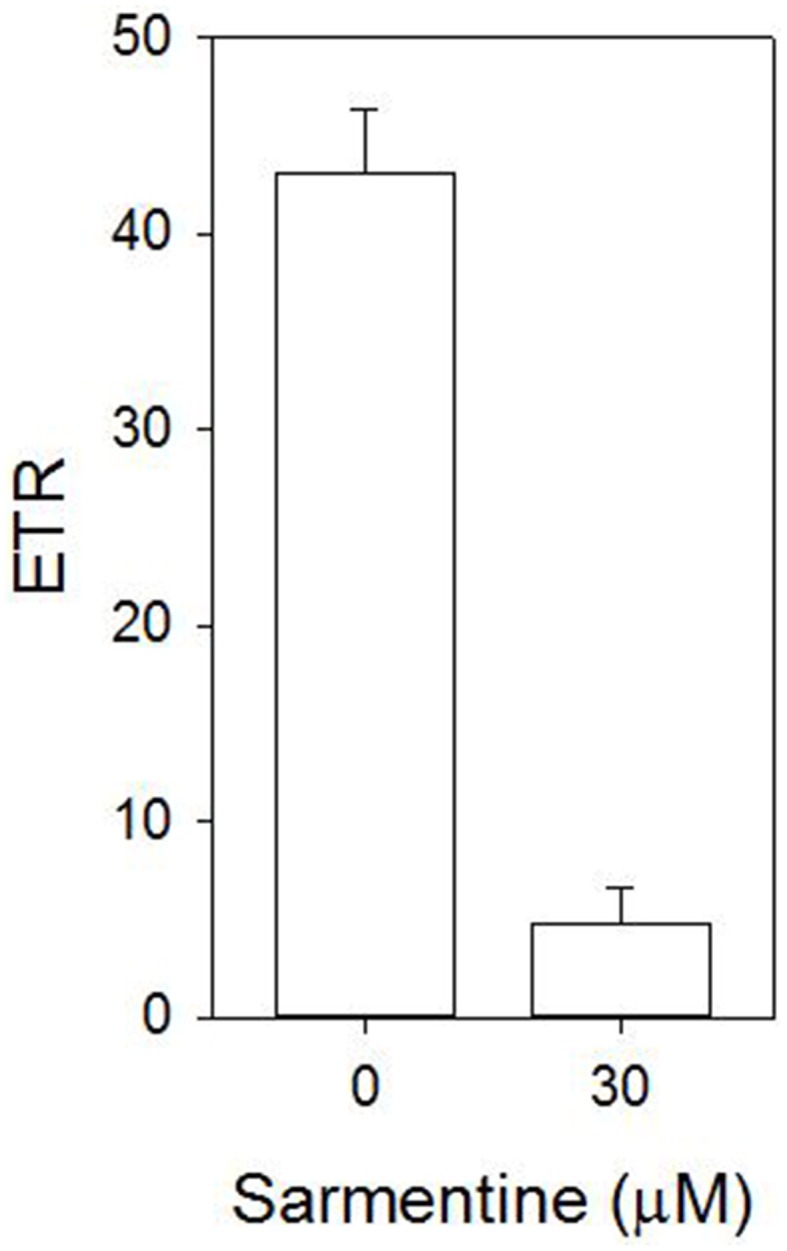
**Effect of sarmentine on photosynthetic efficiency of cucumber cotyledons incubated in darkness for 4 h**.

**Figure 7:**
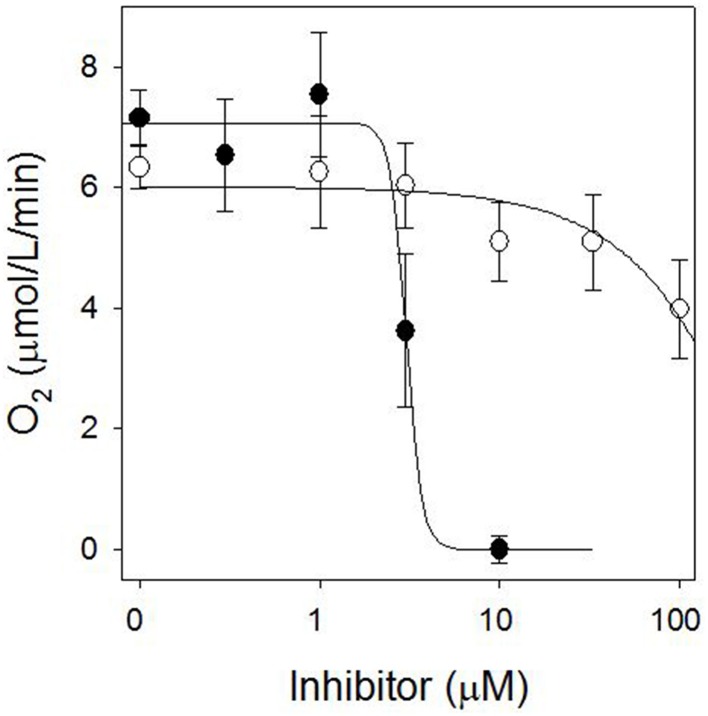
**Effect of sarmentine (●) and pelargonic acid (○) on photosynthetic oxygen evolution in isolated spinach thylakoid membranes**.

The stimulation of electrolyte leakage by light observed at 30 μM sarmentine was accompanied with a strong reduction of photosynthetic electron transport, with only 11% of the total activity after 4 h incubation in the dark (Figure 6). Direct inhibition of photosynthesis was confirmed on isolated thylakoid membranes, where sarmentine caused a rapid and dose-dependent inhibition of oxygen evolution, with an *I*_50_ of 3.0 ± 0.12 μM (Figure 7). On the other hand, pelargonic acid did not inhibit photosynthetic oxygen evolution at concentrations up to 100 μM.

**Figure 8:**
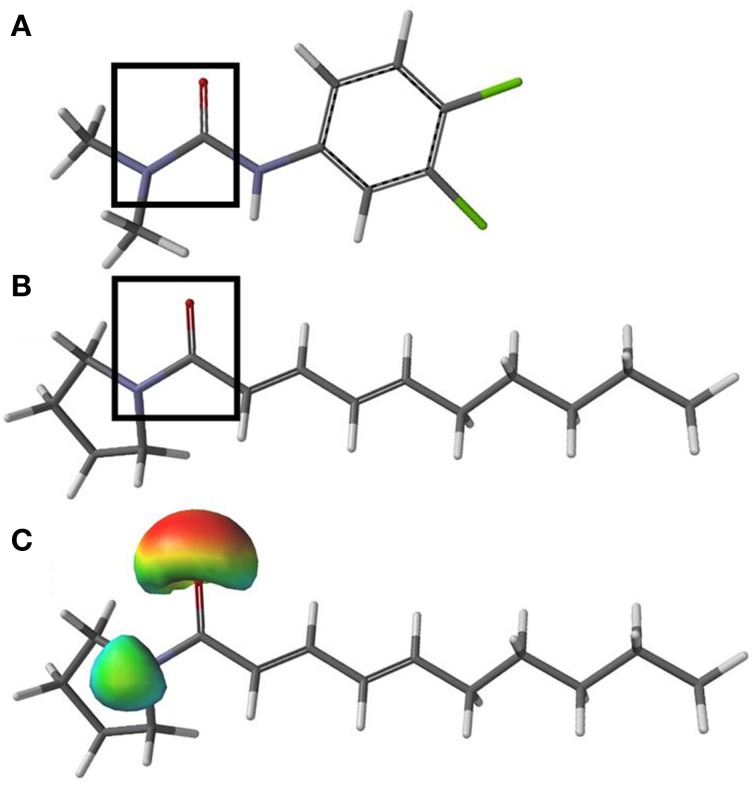
**Minimized structure of diuron (A) and sarmentine (B)**. Black boxes highlight the minimum structural requirements known for “classical” PSII inhibitors. The nitrogen within the boxes carries a positive π-charge, as illustrated in **(C)**, a surface density showing the positive π-charge (blue green) of the nitrogen and negative π-charge (red) of the oxygen atoms. Gray = carbon, white = hydrogen, blue = nitrogen, red = oxygen, and green = chlorine.

**Figure 9:**
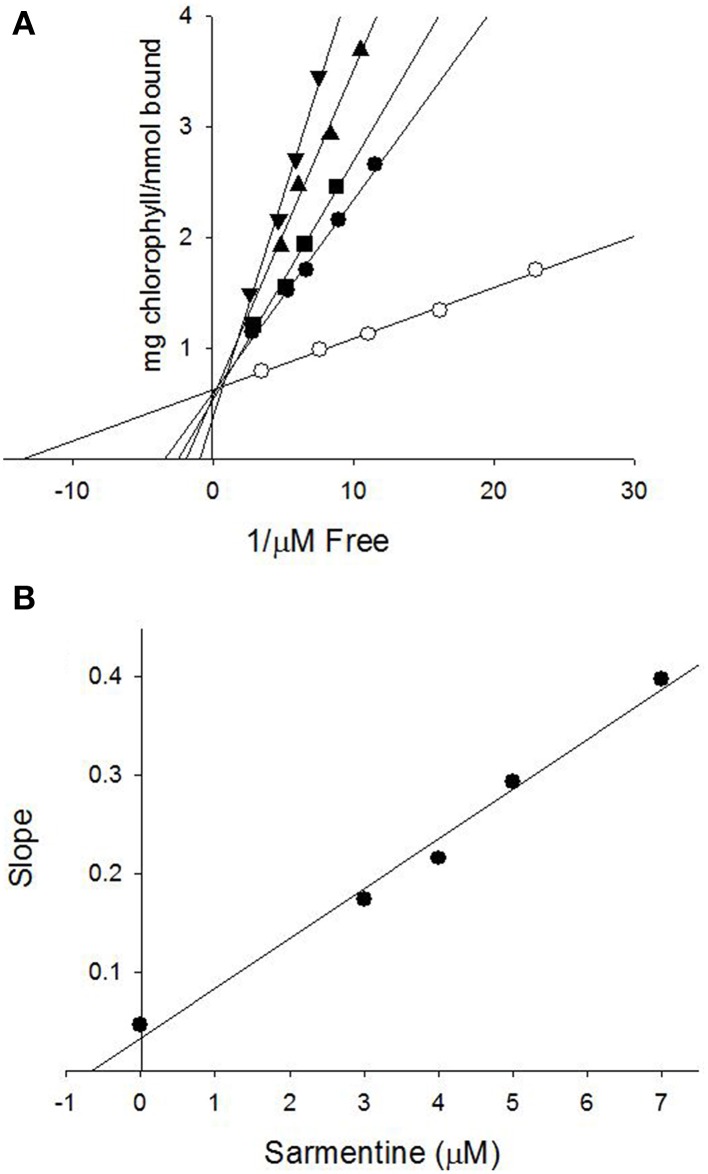
**(A)** Binding of [^14^C]-atrazine to isolated thylakoid membranes and displacement from its binding site by sarmentine. ○ = 0; ● = 3 μM; ■ = 4 μM; ▲ = 5 μM; ▼ = 7 μM sarmentine. **(B)** Estimation of the binding constant of sarmentine on PSII by plotting the concentration of sarmentine vs. the slope of the regression lines in **(A)**.

The ability of sarmentine to inhibit photosynthesis is related to its structural similarity with photosystem II (PSII) inhibitors (Figures 8A,B). In particular, it has an amide group attached to a lipophilic side chain and possesses a nitrogen with a positive π-charge (Figure 8C). These structural features enabled sarmentine to compete with [^14^C]-atrazine for the QB binding site on PSII (Figure 9A). Plotting the slopes of oxygen evolution from the binding kinetic study revealed that sarmentine had an apparent *K*_i_ of 1.5 μM (Figure 9B).

**Figure 10:**
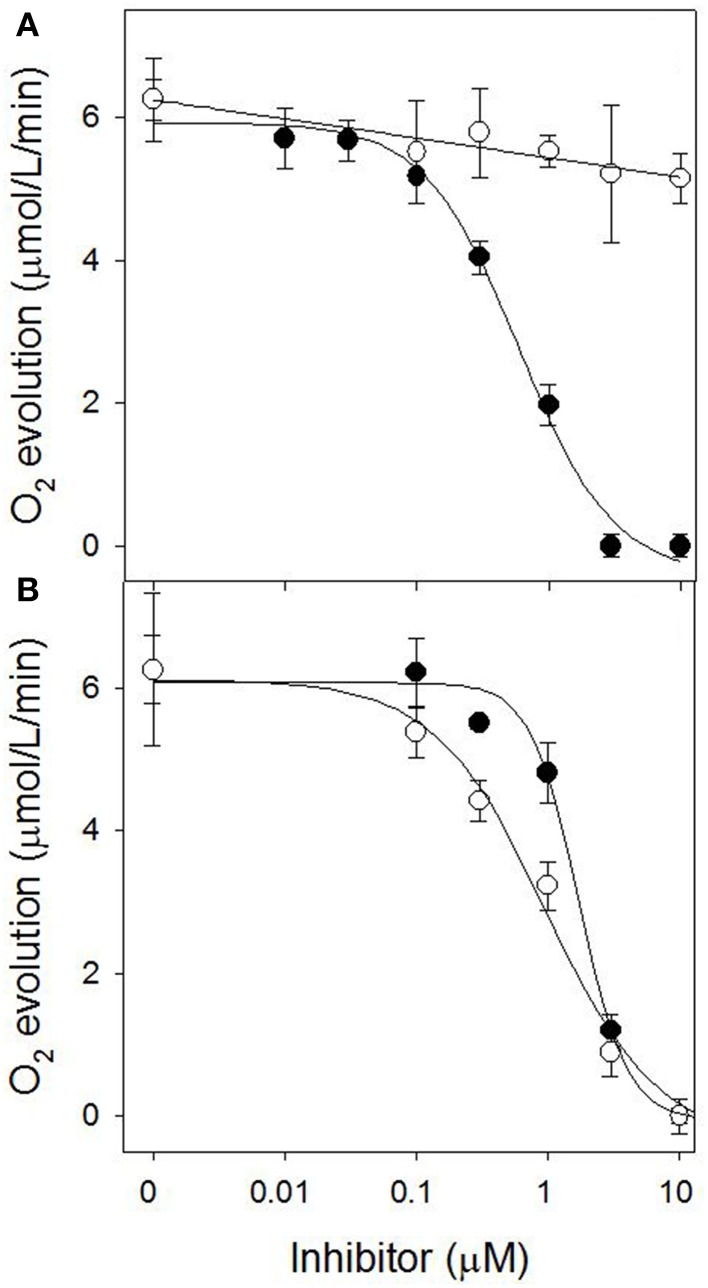
**(A)** Effect of atrazine on O_2_ evolution from thylakoid membrane extracted from susceptible (●) or triazine-resistant (○) pigweed. **(B)** Effect of sarmentine on O_2_ evolution from thylakoid membrane extracted from susceptible (●) or triazine-resistant (○) pigweed.

Since sarmentine acted as a PSII inhibitor, its activity was compared to atrazine on thylakoid membranes isolated from wild-type and triazine-resistant pigweed. As expected, atrazine inhibited oxygen evolution of wild-type pigweed thylakoid preparations (*I*_50_ of 0.60 ± 0.07 μM), but was ineffective (*I*_50_ > 10 μM) on thylakoid preparations from triazine-resistant pigweed (Figure 10A). Sarmentine, on the other hand, inhibited oxygen evolution from both wild-type and triazine-resistant pigweed, with *I*_50_ of 1.72 ± 0.17 and 0.97 ± 0.15 μM, respectively (Figure 10B).

### l-amino acid oxidase assay

The activity of sarmentine was tested on l-amino acid oxidase to be sure that the mechanism of action of this *Piper* amide did not involve indiscriminate enzyme inhibition. l-Amino acid oxidase was selected because this FAD-containing enzyme participates in 8 metabolic pathways (alanine, aspartate, methionine, tyrosine, phenylalanine and tryptophan metabolism, valine, leucine and isoleucine degradation, and alkaloid biosynthesis. Sarmentine did not inhibit l-amino acid oxidase (Supplemental Data 1).

## Discussion

### Alteration of the leaf surface ultrastructure by sarmentine

The appearance of the cuticle and any surface deposit was preserved during the preparation for scanning electron microscopy by avoiding traditional alcohol dehydration and fixation processes. Instead, leaf samples were flash-frozen in nitrogen slush. This process results in ultra-rapid freezing of tissues and prevents the tissue damage associated with the Leidenfrost effect.

The natural appearance of the epidermal cells and cuticular wax layer was preserved in the control samples (Figures 1A,B). The only artifactual features were the slight collapse of the fragile glandular trichomes, but this did not interfere with the interpretation of the micrographs.

The leaf surface of velvetleaf is smooth and void of epicuticular wax crystals (Figure 1), consisting of approximately 36% non-polar and 64% polar lipid components (Harr et al., 1991). Consequently, water droplets have a relatively high contact angle (66°) on the surface of these leaves (Harr et al., 1991). The addition of 0.1% nonionic surfactant dramatically reduces the contact angle, but for the purpose of this study, sarmentine was applied as a 1 mM solution in water with 1% acetone. Though less than ideal for the application of this lipophilic phytotoxin [sarmentine has a log*P* of 3.07 as calculated according to Ghose et al. (1988)], the absence of surfactants or adjuvants ensures that the presence of sarmentine and its effect on the leaf ultrastructure are observed.

Sarmentine residues were visible over the cuticular surface and accumulating as a layer within the grooves of the cells within 1 h of application. No ultrastructural damage is evident within this time frame (Figure 1C), but the collapse of the epidermal cells caused by sarmentine developed within 8 h of application (Figure 1D). This occurs primarily in the vicinity where sarmentine accumulated. However, there was no clear evidence of stripping of the cuticular wax layer, therefore the burn-down symptoms may be due to the interaction of sarmentine with other physiological processes. No effect on the leaf surface was observed in samples treated with the 1% acetone solution (data not shown).

### Destabilization of plasma membrane integrity

The plasma membrane is at the interface between the cell and its environment, and serves to holds the entire cellular structure whole. Consequently, destabilization of the lipid bilayer, often via reactive oxygen species (ROS)-induced stress membrane peroxidation, result in uncontrolled electrolyte leakage and in cell death (McKersie et al., 1990; Mittler, 2002). The possibility that sarmentine affects membrane stability was first tested using a standard cucumber cotyledon disc system (Duke and Kenyon, 1993; Dayan and Watson, 2011) and then applied to several weed species. Sarmentine caused a rapid loss of membrane integrity in all species tested, with *I*_50_ values below 100 μM (Figure 2 and Table 3). A 100 μM concentration corresponds to 22 μg ml^−1^ or 0.0022% sarmentine solutions. This is very active for a natural product, but this does not mean that sarmentine would be active at this concentration in the greenhouse or in the field because the samples are floating on top of the treatment solution in the cotyledon disc assay.

While the concentration required was higher than with sarmentine, pelargonic acid also caused electrolyte leakage, which is consistent with its known mechanism of action (Figure 3 and Table 3) (Dayan and Watson, 2011). Herbicidal organic soaps are based on fatty acid compositions that strip the cuticular wax layer covering the surface of leaves, resulting in rapid loss of membrane integrity. From structure-activity relationship studies, the optimum lipophilic chain length is 8–9 carbons (Lederer et al., 2004; Coleman and Penner, 2006). The contribution of the level of unsaturation is not well known, though some unsaturated fatty acids have been patented as herbicides (Killick et al., 1997). On the other hand, the level of unsaturation of the sarmentine's side chain does not appear to contribute to activity (Huang et al., 2010). From that perspective, sarmentine and pelargonic acid appear to act in a similar manner. However, the light-dependent increase in electrolyte leakage observed at 30 μM sarmentine is not duplicated by pelargonic acid, which would be expected if the two compounds had exactly the same mechanisms of action.

### Inhibition of enoyl-ACP reductase (ENR)

The loss of membrane integrity can be the result of oxidative stress generated by the accumulation of ROS (Dayan and Watson, 2011), but can also result from the inhibition of fatty acid synthesis, as documented with triclosan (McMurry et al., 1998; Heath et al., 1999). In plants, fatty acid biosynthesis is compartmentalized in the chloroplast and catalyzed by a type II fatty acid synthase (FAS). The last step in each elongation cycle is carried out by the enoyl-[ACP]-reductase (ENR), which reduces the dehydrated product of β-hydroxyacyl-[ACP] dehydrase using NADPH or NADH (González-Thuillier et al., 2015). The diphenyl ethers triclosan (a synthetic antimicrobial compound) and cyperin (a fungal metabolite) cause the light-independent loss of membrane integrity by inhibiting ENR (McMurry et al., 1998; Dayan et al., 2008). The possibility that sarmentine also inhibited ENR was explored in part because of the structural similarity between sarmentine and crotonyl-CoA, the substrate of ENR (Figure 5A).

Sarmentine inhibited ENR, with an *I*_50_ = 18.3 ± 4.4 μM, which was much more active than the natural ENR inhibitor cyperin, with an *I*_50_ = 89.0 ± 15.1 μM. This is not a very potent level of activity compared to triclosan, the most common ENR inhibitor, which has an apparent *I*_50_ of 46 ± 5 nM (Dayan et al., 2008). Cyperin and triclosan are diphenyl ethers that are stabilized within the binding pocket of ENR by π-π stacking between one of their phenyl rings and the nicotinamide ring of the NAD^+^ and hydrogen bonding with the side chain of a tyrosine (Heath et al., 1999; Dayan et al., 2008). The binding of sarmentine on ENR is not known.

### Interaction between sarmentine and photosystem II

Sarmentine destabilized plasma membranes at high concentration (100 μM or more), but its activity on electrolyte leakage was minimal at 30 μM (inverted triangle in Figure 2) in darkness. However, its activity increased dramatically upon exposure to light (after the double arrow on Figure 2). This suggests that one of the mechanisms of action of sarmentine involves one of the light-dependent photosynthetic processes.

Photosynthesis is the target of many synthetic herbicides and natural phytotoxins (Draber et al., 1991; Trebst, 2007; Dayan and Duke, 2014). The most common mechanism of action involves inhibition of electron transport on PSII by competing for the binding of plastoquinone on the QB protein. Many of these are typical PSII inhibitors that may or may not compete with the same binding site as atrazine (e.g., sorgoleone, tenuazonic acid) (Einhellig et al., 1993; Czarnota et al., 2001; Chen et al., 2007). However, the other steps of photosynthetic electron transport can be inhibited by natural products as well. For example, stimatellin and certain aurachins interfere with electron transport at the level of cytochrome b6/f complex (Oettmeier et al., 1985, 1990), and the *Streptomyces* phytotoxin pyridazocidin diverts electrons from photosystem I in a similar way as paraquat (Gerwick et al., 1997).

Sarmentine completely halted electron transport at 30 μM after 4 h dark incubation (Figure 4), which is consistent with inhibition of PSII (Dayan and Zaccaro, 2012). Its direct interference of photosynthesis was confirmed by dose-dependent inhibition of oxygen evolution, whereas pelargonic acid was not active (Figure 7). This suggests that the effect on photosynthetic electron transfer is not due to the indirect destabilization of the chloroplast membrane, but rather by direct inhibition of the electron flow in a manner similar to that of diuron or atrazine (Trebst, 2007; Dayan et al., 2010; Dayan and Zaccaro, 2012).

Sarmentine has several of the structural features typical to certain synthetic and natural PSII inhibitors. The minimum structural requirements for these inhibitors include an amide group connected to a lipophilic side chain (Trebst and Draber, 1986). This structural requirement evident in the phenylurea herbicide diuron (Figure 8A) is also present in sarmentine (Figure 8B). Typically, the nitrogen carries a positive π-charge that is important for the binding of the herbicide to the Q_B_ domain of PSII (Ohad and Hirschberg, 1992). Analysis of the partial charge distribution on sarmentine illustrates the presence of such a feature in sarmentine, where the unpaired electrons of the amine are shared with the oxygen of the carbonyl group (Figure 8C).

Final evidence of the effect of sarmentine on PSII consists of its ability to displace atrazine bound to the QB protein (Figure 9A). Taken together, these experiments suggest that sarmentine has a *K*_i_ of 1.5 μM, which is better than some other natural products like tenuazonic acid at 147 μM (Chen et al., 2008) but not as good as most synthetic herbicides (Tischer and Strotmann, 1977). Interestingly, sarmentine inhibited the electron flow from thylakoid membranes isolated from triazine-resistant pigweed, whereas atrazine was only active on the wild-type pigweed (Figure 10). This is similar to what has been reported with other natural PSII inhibitors. There are two known binding sites on the Qb protein (Oettmeier et al., 1982). The Ser264 family (also called the classical family) is important for the binding of triazine-type inhibitors, whereas the His215 family (also called the quinone or phenolic family) is important for the binding of quinone-type inhibitors (Dayan et al., 2009a). Consequently, a mutation of Ser264 to Gly or Ala causes resistance to triazines, but not to other inhibitors (Dayan et al., 2009a).

In conclusion, application of sarmentine results in rapid desiccation of the foliage but its herbicidal activity is more complex than the physical removal of cuticle associated with organic soap herbicides (Coleman and Penner, 2006, 2008). Indeed, the loss of plasma membrane integrity is the consequence of at least two mechanisms of action. Sarmentine crosses the cuticle and penetrates the cells where it interferes with fatty acid synthesis by inhibiting ENR and with photosynthesis by competing for the binding of plastoquinone on PSII.

### Conflict of interest statement

The authors declare that the research was conducted in the absence of any commercial or financial relationships that could be construed as a potential conflict of interest.
