# Supplementary figures and images for: Sarmentine, a natural herbicide from Piper species with multiple herbicide mechanisms of action
Source: Front Plant Sci. 2015 Apr 8;6:222. doi: 10.3389/fpls.2015.00222 (PMC4389368; doi:10.3389/fpls.2015.00222)

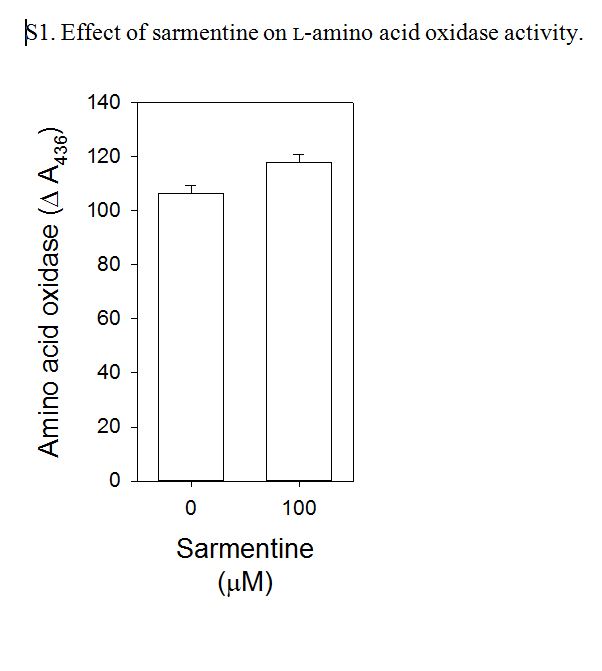

Supplement: Supplementary file 1 [file Image1.JPEG]
